# SIRT1 activation by 2,3,5,6-tetramethylpyrazine alleviates neuroinflammation via inhibiting M1 microglia polarization

**DOI:** 10.3389/fimmu.2023.1206513

**Published:** 2023-08-04

**Authors:** Yu Chen, Fu Peng, Chao Yang, Huan Hou, Ziwei Xing, Junren Chen, Li Liu, Cheng Peng, Dan Li

**Affiliations:** ^1^ State Key Laboratory of Southwestern Chinese Medicine Resources, School of Pharmacy, Chengdu University of Traditional Chinese Medicine, Chengdu, China; ^2^ Department of Pharmacology, Key Laboratory of Drug-Targeting and Drug Delivery System of the Education Ministry, Sichuan Engineering Laboratory for Plant-Sourced Drug and Sichuan Research Center for Drug Precision Industrial Technology, West China School of Pharmacy, Sichuan University, Chengdu, China; ^3^ National Engineering Research Center for Marine Aquaculture, Institute of Innovation and Application, Zhejiang Ocean University, Zhoushan, Zhejiang, China; ^4^ Chiatai Qingchunbao Pharmaceutical Co., Ltd., Hangzhou, China

**Keywords:** 2, 3, 5, 6-tetramethylpyrazine, neuroinflammation, microglia polarization, SIRT1, NF-κB

## Abstract

**Background:**

Neuroinflammation has been reported as a potential contributing factor to brain diseases, and is characterized by activated microglia with release of multiple inflammatory mediators. 2,3,5,6-Tetramethylpyrazine (TMP) is an active alkaloid in *Ligusticum chuanxiong* Hort. and has various biological activities, including anti-inflammatory and neuroprotection properties. However, the anti-neuroinflammatory activity of TMP has been less studied and its potential molecular mechanisms in this field remain unclear. This study aimed to investigate the effects of TMP and its underlying mechanisms in neuroinflammation.

**Methods:**

*In vitro*, lipopolysaccharide (LPS)-stimulated BV2 microglia were used to assess the effects of TMP on inflammatory cytokines as well as the components of the SIRT1/NF-κB signaling pathway, which were measured by using ELISA, western blotting, qRT-qPCR and immunofluorescence. Moreover, LPS-induced acute neuroinflammation model in mice was performed to detect whether TMP could exert anti-neuroinflammatory effects *in vivo*, and the EX527, a SIRT1 inhibitor, were given intraperitoneally every two days prior to TMP treatment. Serums and spinal trigeminal nucleus (Sp5) tissues were collected for ELISA assay, and the Sp5 tissues were used for HE staining, Nissl staining, immunofluorescence, qRT-PCR and western blotting.

**Results:**

*In vitro*, TMP treatment significantly reduced the secretion of pro-inflammatory cytokines, including TNF-α and IL-6, promoted SIRT1 protein expression and inactivated NF-κB signaling pathway in LPS-induced neuroinflammation. Interestingly, pretreatment with EX527 blocked the therapeutic effects of TMP on neuroinflammation *in vitro*. Furthermore, TMP reduced the levels of pro-inflammatory cytokines and chemokines, and prevented microglia from polarizing towards a pro-inflammatory state through activating SIRT1 and inhibiting NF-κB activation in LPS-induced neuroinflammation in mice. And EX527 reversed the beneficial effects of TMP against LPS exposure in mice.

**Conclusion:**

In summary, this study unravels that TMP could mitigate LPS-induced neuroinflammation via SIRT1/NF-κB signaling pathway.

## 1 Introduction

Neuroinflammation, an inflammatory response against to various endogenous or exogenous stimuli in the central nervous system (CNS), acts as a common denominator of almost all brain disorders, including neurodegenerative, neuropsychiatric and cerebrovascular diseases such as Alzheimer’s disease, depression and migraine (1–4), manifested by a particularly high incidence in the spinal trigeminal nucleus (Sp5) region (5, 6). Immune cells, like microglia, as well as molecular components, such as cytokines and chemokines, are key regulators of neuroinflammation, while dysregulated activity of the above cellular and molecular elements lead to inappropriate inflammatory responses that might cause tissue damage and influence CNS functions (7, 8). Thus, treatment targeting neuroinflammation represents an exciting novel neuroprotective strategy.

Microglia are the major intrinsic immune cells in the CNS, and their inflammatory response is a critical mediator in the pathogenesis and progression of brain diseases (9). According to the predominance of secreted factors, activated microglia have been classified into two phenotypes, pro-inflammatory microglia (M1) and anti-inflammatory microglia (M2), respectively. M1 microglia generate pro-inflammatory cytokines and highly express iNOS, COX2 and CD86; while M2 microglia with the cell marker of CD206 and Arg-1, produce anti-inflammatory cytokines (10). Besides, numerous studies have found that microglial polarization toward M1 state accompany by the activation of NF-κB pathway, in turn, blocking NF-κB signals could suppress M1 polarization and reduce the secretion of pro-inflammatory mediators in CNS diseases (11, 12). Hence, interventions to modulate the phenotype of microglia may be a promising approach for the treatment of inflammation-associated brain diseases.

2,3,5,6-Tetramethylpyrazine (TMP), as one of the main active components of *Ligusticum chuanxiong* Hort., has been demonstrated to exert neuroprotective effects (13). A study showed that TMP could reduce the permeability of the blood-brain barrier and increase tight junction proteins expression to promote neurological recovery after ischemia/reperfusion injury (14). Moreover, TMP also inhibited neuronal apoptosis via inhibiting the mitochondria-related Bax/Bcl-2 and Caspase-3 pathway in rats with vascular dementia (15). However, at present, the role of TMP in neuroinflammation and associated brain diseases is poorly understood. Our study was designed to evaluate the roles and mechanisms of TMP in lipopolysaccharide (LPS)-induced acute neuroinflammation of mice and to provide a new candidate drug or lead compound to treat inflammation-related disorders in CNS.

## 2 Materials and methods

### 2.1 Cell culture

BV2 microglia cells from Procell (China) were grown in MEM (Procell, China) with 10% FBS (Gibco, USA) at 37 °C and 5% CO_2_ in a humidified air environment.

### 2.2 Animals and protocols

All *in vivo* experiments were approved by the Animal Ethics Committee of Chengdu University of Traditional Chinese Medicine (No. 2021-30). 20 male C57BL/6 mice (18-22g), provided by SPF Biotechnology Co., Ltd (No. SCXK 2019-0010, Beijing, China), were maintained at 22 ± 2°C temperatures and 55 ± 5% relative humidity under a 12 h light/dark cycle with free access to food and water *ad libitum*. A mouse model of acute neuroinflammation was established by a single injection of LPS as described previously (16). Upon adaptive feeding for one week, the mice were randomly divided into four groups (n=5): Control group; LPS group; TMP + LPS group; and TMP+EX527+LPS group. TMP+LPS and TMP+EX527+LPS group were injected intraperitoneally (i.p.) with 50 mg/kg TMP (Chengdu Must Bio-technology CO., Ltd, China) that was dissolved in saline with 20% PEG 300. Control and LPS group were treated with (i.p.) saline containing 20% PEG 300 once a day for consecutive 14 days. Starting from the 8th day, mice in the TMP+EX527+LPS group were i.p. with 5 mg/kg EX527 (Selleck, USA, dissolved in DMSO: PEG 300: saline, 5:30:65) every two days for a total of four times. On the 15th day, the LPS, TMP+LPS and TMP+EX527+LPS group were injected i.p. with 5 mg/kg LPS (Escherichia coli 0127: B8, Sigma-Aldrich, USA), and Control group were treated (i.p.) with saline. After 6 hours of LPS injection, the mice were anesthetized by inhalation with isoflurane and the blood and brains were collected.

### 2.3 MTT assay

BV2 cells were inoculated into 96-well plates (5×10^3^ cells/well). After 24 h, TMP with or without LPS at different concentrations was used to treat the cells for 24 h. MTT assay was used to determine cell viability as described previously (17).

### 2.4 Measurement of cytokine levels

Concentrations of TNF-α and IL-6 in the cell medium and the serum and Sp5 tissues from LPS-induced mice were measured by ELISA kits (Neobioscience, China).

### 2.5 qRT-qPCR analysis

Total RNA from BV2 cells and Sp5 tissues were derived with TRIzol Reagent (Invitrogen, USA) and reversed to cDNAs using RevertAid Master Mix with DNase I (Thermo Fisher Scientific, USA). cDNA samples, gene specific primers (**Table 1**) and SYBR™ Green Master Mix (Thermo Fisher Scientific) were used to perform qPCR experiments according to the protocol, which were collected by ABI StepOnePlus PCR system (ABI-7500, Thermo Fisher Scientific). The relative mRNA expressions of genes were calculated by the 2^-△△CT^ method as described previously (18).

**Table 1:** List of oligonucleotide primer pairs used in qRT-PCR.

Gene	Forward	Reverse
*18S*	AGCCTGCGGCTTAATTTGAC	CAACTAAGAACGGCCATGCA
*TNF-α*	CCCCAAAGGGATGAGAAGTTC	CCTCCACTTGGTGGTTTGCT
*IL-6*	CCAGAAACCGCTATGAAGTTCC	GTTGGGAGTGGTATCCTCTGTGA
*IL-1β*	GTTCCCATTAGACAACTGCACTACAG	GTCGTTGCTTGGTTCTCCTTGTA
*iNOS*	GAACTGTAGCACAGCACAGGAAAT	CGTACCGGATGAGCTGTGAAT
*COX-2*	CAGTTTATGTTGTCTGTCCAGAGTTTC	CCAGCACTTCACCCATCAGTT
*CD86*	TTGTGTGTGTTCTGGAAACGGAG	AACTTAGAGGCTGTGTTGCTGGG
*Arg-1*	GTGAAGAACCCACGGTCTGT	GCCAGAGATGCTTCCAACTG
*CD206*	CTTCGGGCCTTTGGAATAAT	TAGAAGAGCCCTTGGGTTGA
*Mcp-1*	GGATCGGAACCAAATGAGAT	ATTTACGGGTCAACTTCACA
*Ccl-3*	CAGCCAGGTGTCATTTTCCT	CAGGCATTCAGTTCCAGGTC
*Ccl-5*	CTACTGCTTTGCCTACCTCT	ACACACTTGGCGGTTCCTT
*Cxcl10*	TTCTGCCTCATCCTGCTG	AGACATCTCTGCTCATCATTC

### 2.6 Western blot analysis

As described previously (19), proteins in BV2 cells and Sp5 tissues were extracted using RIPA buffer with protease inhibitor and PMSF (Beyotime, China). BCA protein assay kit (Thermo Fisher Scientific) was used to measure the protein concentration. Equal amounts of proteins sample (10-20 µg) were fractionated on SDS-PAGE and shifted onto PVDF membranes (Bio-Rad, USA). The membranes were blocked with 5% skimmed milk for 1 h and then incubated with primary antibodies (**Table 2**) overnight at 4°C. After that, the blots were incubated with secondary antibody for 2 h at room temperature. The immune-blotting signals were visualized by chemiluminescence (Thermo Fisher Scientific) using Tanon 5200 (China). Quantitative analysis was analyzed by Image J software.

**Table 2:** Antibodies used in Western blot and immunofluorescence.

Antibody	Source	Vendor	Catalog No.
anti-phospho-p65 (Ser536)	Rabbit	Cell Signaling Technology	#3033
anti-p65	Rabbit	Cell Signaling Technology	#8242
anti-phospho-IKKα/β (Ser176/180)	Rabbit	Cell Signaling Technology	#2697
anti-IKKα	Rabbit	ABclonal	A19694
anti-IKKβ	Rabbit	Cell Signaling Technology	#8943
anti-phospho-IκBα (Ser32)	Rabbit	Cell Signaling Technology	#2859
anti-IκBα	Mouse	Cell Signaling Technology	#4814
anti-SIRT1	Mouse	Cell Signaling Technology	#8469
α-Tubulin	Mouse	ABclonal	AC012
anti-Iba-1	Rabbit	ABclonal	A20844
anti-CD206	Mouse	Santa Cruz Biotechnology	sc-58986
anti-iNOS	Mouse	Santa Cruz Biotechnology	sc-7271
anti-CD86	Mouse	Santa Cruz Biotechnology	sc-28347
Anti-rabbit IgG, HPR-linked Antibody	Goat	Cell Signaling Technology	#7074
Anti-mose IgG, HPR-linked Antibody	Horse	Cell Signaling Technology	#7076
Alexa Flour 488 goat anti-mouse IgG	Goat	Thermo Fisher Scientific	A11029
Alexa Flour 594 goat anti-Rabbit IgG	Goat	Thermo Fisher Scientific	A11037

### 2.7 NF-κB immunofluorescence

BV2 cells were fixed in 4% paraformaldehyde for 10 min, infiltrated with 0.5% Triton X-100 (Bio-Rad) for 20 min after washing 3 times with PBS and then incubated with anti-p65 antibody (1:400) at 4°C overnight, followed by Alexa Fluor 488 goat anti-rabbit IgG (1:1000) for 1 h and DAPI (1:1000, Beyotime, China) for 20 min. Images were acquired with a confocal microscope (FV-OSR, Olympus, Japan) and quantitated using ImageJ.

### 2.8 HE staining and Nissl staining

After the brain tissue was fixed with 4% paraformaldehyde and embedded in paraffin, 5 μm sections were cut for HE and Nissl staining (20). The images were obtained by an optical microscope (Ti2, Nikon, Japan) and quantitated using ImageJ.

### 2.9 Immunofluorescence analysis

As described previously (21), after deparaffinizing and rehydrating, the 5 μm sections of paraffin-embedded brain tissue were soaked with 10 mM citric acid (pH 6.0) and heated in a water bath to recover antigenicity, and then blocked with 10% BSA at room temperature for 1h. After that, sections were stained with primary anti-Iba-1 (1:200) and anti-CD206 (1:200) antibodies, anti-iNOS (1:200) antibodies, and anti-CD86 (1:200) antibodies at 4°C overnight, followed by fluorescently labeled secondary antibodies for 1 h and DAPI for another 20 min. A confocal microscope (FV-OSR, Olympus, Japan) was used to acquire images of the stained sections. The images were quantitated using ImageJ.

### 2.10 Statistical analyses

All data were analyzed using GraphPad Prism 9.4.0 software and were presented as mean ± SD. The differences between groups were performed by one-way ANOVA, and *P* < 0.05 were considered statistically significant.

## 3 Results

### 3.1 TMP ameliorates LPS-stimulated neuroinflammation in BV2 cells

**Figure 1:**
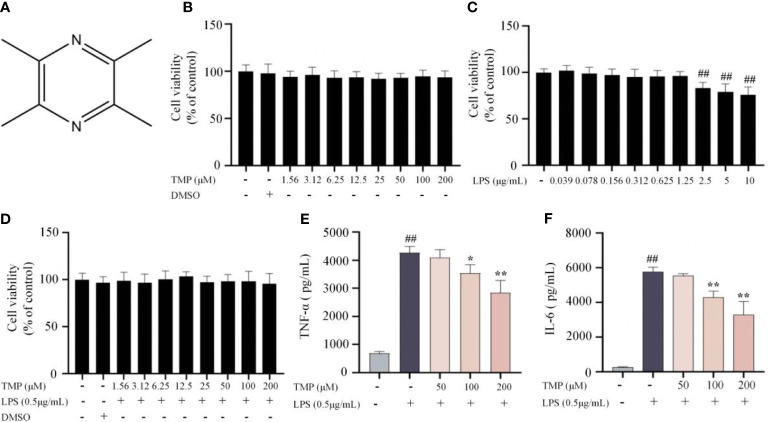
TMP ameliorated neuroinflammatory responses in LPS-stimulated BV2 cells. **(A)** Structure of TMP. **(B)** BV2 cells were treated with TMP at different concentrations range from 1.56-200 μM for 24 h, and cell viability was determined using MTT assay (n=18). **(C)** BV2 cells were treated with LPS (0.039-10 μg/mL) for 24 h, and cell viability was determined using MTT assay (n=18). **(D)** BV2 cells were with TMP (1.56-200 μM) with LPS (0.5 μg/mL) for 24 h, and cell viability was measured (n=18). **(E, F)** BV2 cells were pretreated with 50, 100, and 200 μM TMP for 4 h and then stimulated by LPS (0.5 μg/mL) for 18 h The levels of TNF-α **(E)** and IL-6 **(F)** were determined by ELISA kits (n=3). Data are expressed as mean± SD. ^##^ *P* < 0.01 *vs*. Control, ^*^ *P* < 0.05 vs. LPS, ^**^ *P* < 0.01 vs. LPS.

Firstly, the cytotoxicity of different concentrations of TMP (structure in **Figure 1A**) on BV2 cells was assessed by MTT assay. Results showed that TMP at 0-200 μM had no significant observable effect on cell viability with or without the presence of 0.5 μg/mL LPS (**Figures 1B–D**). Next, we assessed whether TMP exerted inhibitory effect on inflammatory cytokines in LPS-stimulated BV2 cells. TMP significantly restrained the production of TNF-α and IL-6 according to ELISA results (**Figures 1E, F**). Thus, TMP alleviates the LPS-induced neuroinflammatory response in BV2 cells.

### 3.2 TMP suppresses LPS-stimulated neuroinflammation via NF-κB signals

**Figure 2:**
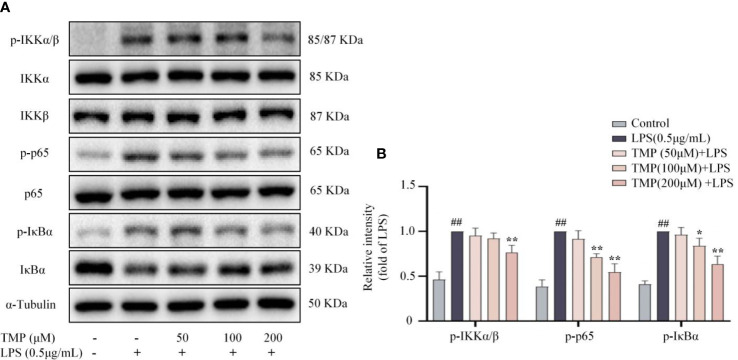
TMP inhibited NF-κB signaling pathway in LPS-induced BV2 cells. Cells were pretreated with 50, 100, and 200 μM TMP for 4 h and then stimulated with LPS (0.5 μg/mL) for 1h. **(A)** Representative images of blot for p-IKKα/β, IKKα, IKKβ, p-IκBα, IκBα, p-p65 and p65. **(B)** Data are normalized to the mean value of LPS group, and quantitative analysis of p-IKKα/β/IKKα/IKKβ, p-IκBα/IκBα, p-p65/p65 were detected by Image J Data are expressed as mean± SD, n=3. ^##^ *P <* 0.01 vs. Control, ^*^ *P <* 0.05 vs. LPS, ^**^ *P <* 0.01 vs. LPS.

According to recent studies, NF-κB signaling plays crucial roles in modulating neuroinflammation (22). LPS stimulation promoted phosphorylation of IKKα/β, p65 and IκBα, and TMP inhibited the phosphorylation of the above proteins (**Figures 2A, B**). Thus, TMP could suppressed the activation of NF-κB pathway.

### 3.3 TMP alleviates neuroinflammation through promoting SIRT1

**Figure 3:**
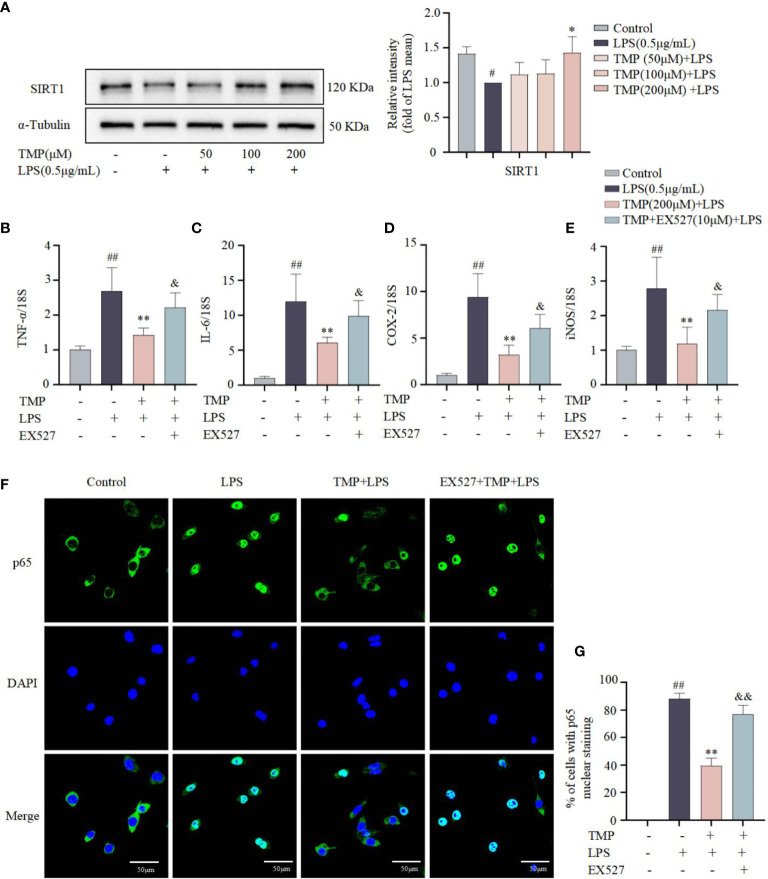
The anti-neuroinflammatory effects of TMP in LPS-induced BV2 cells were partially mediated by SIRT1. **(A)** BV2 cells were pretreated with 50, 100, and 200 μM TMP for 4 h and then stimulated with LPS (0.5 μg/mL) for 18 h. The protein expression of SIRT1 was determined by Western blotting (n=3). Data were normalized to the mean value of the LPS group. **(B–E)** SIRT1 inhibitor EX527 (10 μM) was given to BV2 cells 0.5 h prior to TMP (200 μM) treatment, 4 h later, BV2 cells were stimulated with LPS (0.5 μg/mL) for 12 h. The mRNA levels of TNF-α **(B)**, IL-6 **(C)**, COX-2 **(D)** and iNOS **(E)** were analyzed by qRT-PCR, and normalized to 18S (n=6). **(F)** BV2 cells were treated with TMP (200 μM) or EX527 (10 μM) + TMP (200 μM) for 4 h and subsequently induced with LPS (0.5 μg/mL) for 1 h. Representative immunofluorescence staining for p65 in the different groups. Nuclei were stained with DAPI. **(G)** The percentage of cells with p65 nuclear staining was evaluated from the immunostaining images and analyzed by Image J (n=6). Data are expressed as mean± SD. ^#^ *P* < 0.05 vs. Control, ^##^ *P* < 0.01 vs. Control, ^*^ *P* < 0.05 vs. LPS, ^**^ *P* < 0.01 vs. LPS, &*P* < 0.05 vs. TMP+LPS, &&*P* < 0.01 vs. TMP+LPS.

SIRT1 negatively regulate the activity of NF-κB signaling pathway in the model of neuroinflammation (23). The expression of SIRT1 protein was decreased after LPS stimulation, and TMP significantly increased SIRT1 expression in LPS-induced BV2 cells (**Figure 3A**). In order to further detect whether the anti-neuroinflammatory effect of TMP is mediated by SIRT1, the SIRT1 inhibitor EX527 was used. As shown in **Figures 3B–E**, in LPS-stimulated BV2, TMP reduced the transcription of TNF-α, IL-6, COX-2 and iNOS, while EX527 reversed the effects of TMP on these mRNA expressions. Since p65 is a key nuclear transcription factor in the NF-κB pathway and its nuclear translocation is critical to the activation of the NF-κB pathway (24), subsequently we explored whether TMP affects the translocation of p65. In LPS-induced BV2 cells, the proportion of cells with p65 translocated to the nucleus was increased, TMP treatment reduced nuclear accumulation of p65, while EX527 reversed this change (**Figures 3F, G**). These data suggest that SIRT1 mediates, at least to some extent, the anti-neuroinflammatory effects of TMP in LPS-induced BV2 cells.

### 3.4 TMP ameliorates neuroinflammatory responses in LPS-induced mice

**Figure 4:**
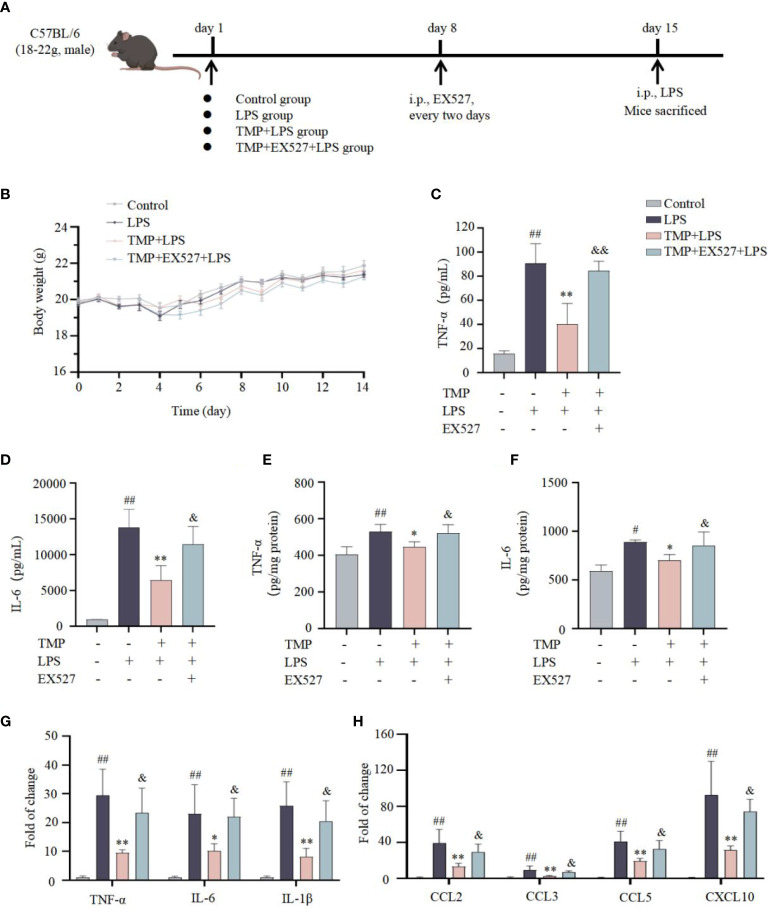
TMP ameliorated neuroinflammatory responses in LPS-induced mice. **(A)** Establishment of a mouse model of acute neuroinflammation. **(B)** Body weight in each group of mice (n=5). **(C, D)** The levels of TNF-α **(C, E)** and IL-6 **(D, F)** in serum **(C, D)** and Sp5 tissues **(E, F)** were determined by ELISA kits (n=5). **(G, H)** qRT-qPCR analysis of cytokines **(G)** and chemokines **(H)** in the Sp5 tissues of mice (n=5). Data are expressed as mean± SD. ^#^ *P* < 0.05 vs. Control, ^##^ *P* < 0.01 vs. Control, ^*^ *P* < 0.05 vs. LPS, ^**^ *P* < 0.01 vs. LPS, &*P* < 0.05 vs. TMP+LPS, &&*P* < 0.01 vs. TMP+LPS.

**Figure 5:**
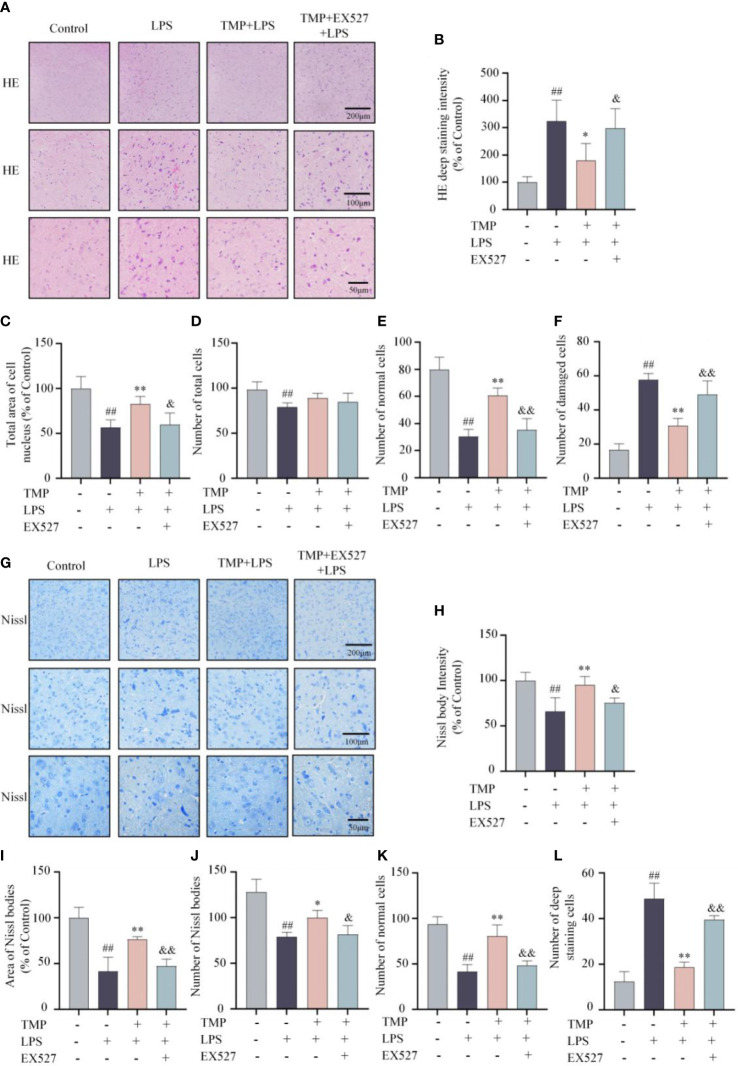
TMP mitigated LPS-induced lesion in the Sp5 of brain tissue in mice. **(A)** Representative HE staining images of Sp5. **(B–F)** Quantification of HE staining, including HE deep staining intensity **(B)**, total area of cell nucleus **(C)**, number of total cells **(D)**, number of normal cells **(E)** and number of damaged cells **(F)**. **(G)** Representative Nissl staining images of Sp5. **(H–L)** Quantification of Nissl staining, including Nissl body intensity **(H)**, area of Nissl bodies **(I)**, number of Nissl bodies **(J)**, number of normal cells **(K)** and number of deep staining cells **(L)**. Data are expressed as mean± SD, n=5. ^#^ *P* < 0.05 vs. Control, ^##^ *P* < 0.01 vs. Control, ^*^ *P* < 0.05 vs. LPS, ^**^ *P* < 0.01 vs. LPS, &*P* < 0.05 vs. TMP+LPS, &&P < 0.01 vs. TMP+LPS.

Next, to investigate the effects of TMP on neuroinflammation *in vivo*, LPS induced acute neuroinflammation model was established (**Figure 4A**). TMP had no significant effect on the body weight of mice (**Figure 4B**). Following LPS stimulation, the contents of TNF-α and IL-6 in serum and Sp5 tissues were significantly increased, whereas TMP administration reduced these cytokines levels (**Figures 4C–F**). Meanwhile, the results of qRT-PCR indicated that TMP treatment markedly decreased the expressions of cytokines, like TNF-α, IL-6 and IL-1β, as well as chemokines, including CCL2, CCL3, CCL5, and CXCL10 in the Sp5 of brain tissue from LPS-induced mice (**Figures 4G, H**). Additionally, data of HE staining and Nissl staining of Sp5 tissues showed that LPS administration caused obvious neuronal loss and morphological changes, including nuclear condensation, hyperchromatism and obvious eosinophilic cytoplasmic degeneration, while TMP ameliorated these pathological changes (**Figure 5**). Notably, SIRT1 inhibitor EX527 reversed these effects of TMP (**Figures 4**, **5**).

### 3.5 TMP regulates polarization of microglia in LPS-induced mice

**Figure 6:**
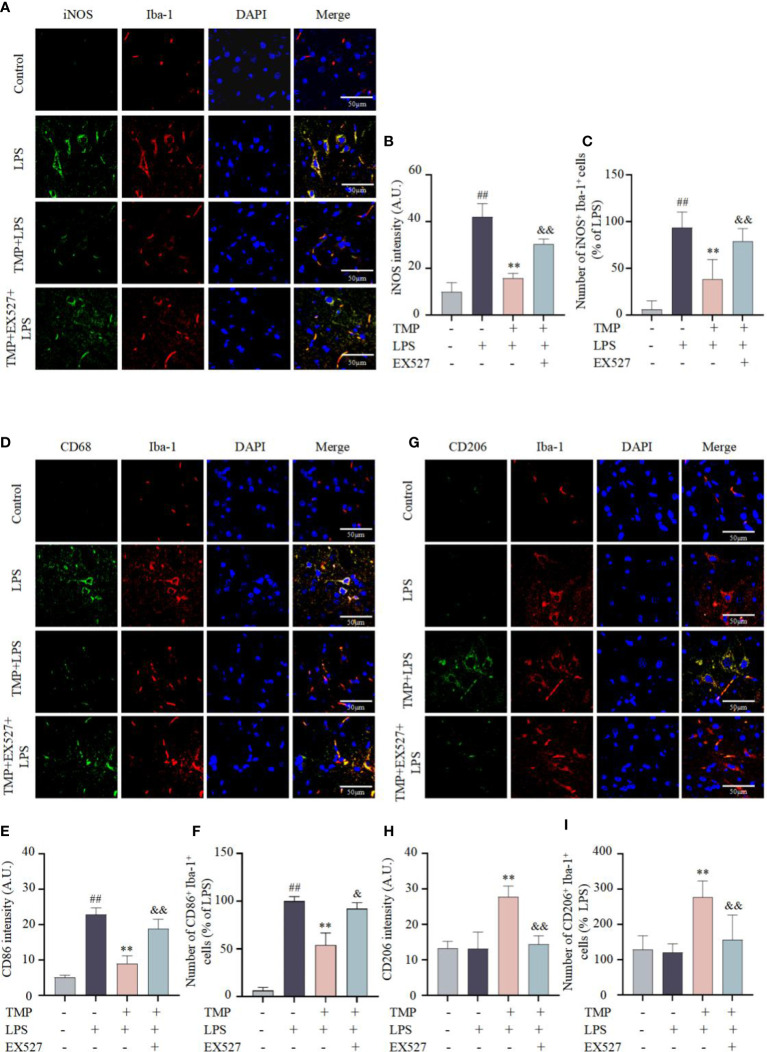
TMP modulated the microglial M1/M2 polarization in LPS-induced mice. **(A)** Representative immunofluorescence staining images of M1 microglia markers iNOS (green) and Iba-1 (red) in the Sp5 of LPS-induced mice **(B, C)** Quantification of iNOS intensity **(B)** and the number of iNOS^+^Iba-1^+^cells **(C)**. **(D)** Representative immunofluorescence staining images of M1 microglia markers CD86 (green) and Iba-1 (red) in the Sp5 of LPS-induce mice. **(E, F)** Quantification of CD86 intensity **(E)** and the number of CD86^+^Iba-1^+^cells **(F)**. **(G)** Representative immunofluorescence staining images of M2 microglia markers CD206 (green) and Iba-1 (red) in the Sp5 of LPS-induce mice. **(H, I)** Quantification of CD206 intensity **(H)** and the number of CD206^+^Iba-1^+^cells **(I)**. Data are expressed as mean± SD, n=5. ^##^ *P* < 0.01 vs. Control, ^**^ *P* < 0.01 vs. LPS, &*P* < 0.05 vs. TMP+LPS, &&*P* < 0.01 vs. TMP+LPS.

**Figure 7:**
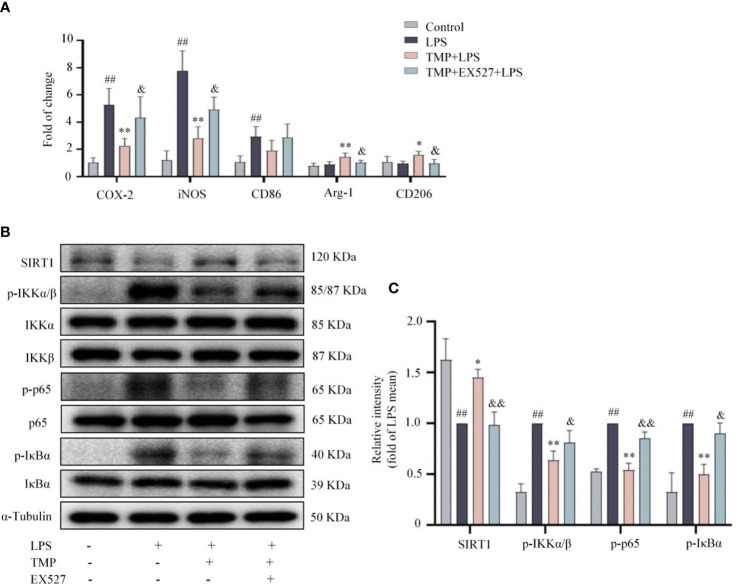
TMP prevented neuroinflammation *via* SIRT1/NF-κB pathway in LPS-treated mice. **(A)** RT-qPCR analysis of M1 microglia markers (COX2, iNOS, and CD86) and M2 microglia markers (Arg-1 and CD206) in the Sp5 tissue of mice. **(B)** Representative blots of SIRT1, p-IKKα/β, IKKα, IKKβ, p-IκBα, IκBα, p-p65 and p65 in the Sp5 tissue of mice. α-Tubulin was used as an internal loading control. **(C)** Data are normalized to the mean value of LPS group, and quantitative analysis of SIRT1, p-IKKα/β, p-IκBα, p-p65 was measured by Image J. Data are expressed as mean± SD, n=5. ^##^ *P* < 0.01 vs. Control, ^*^ *P* < 0.05 vs. LPS, ^**^ *P* < 0.01 vs. LPS, &*P* < 0.05 vs. TMP+LPS, &&*P* < 0.05 vs. TMP+LPS.

Activation and polarization of microglia is one of the characteristics of neuroinflammation (25). To identify whether the anti-neuroinflammatory effects of TMP was related to microglia polarization, immunofluorescence staining and qRT-qPCR were performed to evaluate microglia markers in the Sp5 tissue. Results indicated that compared with LPS group, TMP treatment suppressed the expressions of M1 markers iNOS (**Figures 6A–C**) and CD86 (**Figures 6D–F**), but facilitated the expression of M2 marker CD206 (**Figures 6G–I**). Additionally, the mRNA expression of M1 marker COX-2, iNOS and CD86 (**Figure 7A**) were significantly increased after LPS injection, while TMP inhibited the expression of M1 markers and increased the transcription of the M2 markers, including Arg-1 and CD206 (**Figure 7A**) in Sp5 tissue. And EX527 partially counteracted the effects of TMP on microglia polarization (**Figures 6**, **7A**). These data suggests that TMP could modulated the microglial M1/M2 polarization in acute neuroinflammation, partly mediated by SIRT1.

### 3.6 TMP promoted SIRT1 expression and suppressed NF-κB signals in LPS-induced mice

SIRT1 expression in the Sp5 was observably decreased after LPS injection, and TMP markedly reversed the reduction of SIRT1 (**Figures 7B, C**). Besides, after LPS injection, the expressions of p-IKKα/β, p-p65and p-IκBα were elevated, and TMP treatment notably prevented the changes. Consistently, the effects of TMP were partially blocked by EX527 (**Figures 7B, C**). These data suggest that TMP ameliorates the neuroinflammatory response induced by LPS through SIRT1/NF-κB signaling pathways.

## 4 Discussion

Neuroinflammation, pervasive immune response occurred under brain disorders, is characterized by over-activated microglia with massive secretion of pro-inflammatory mediators, like cytokines, chemokines as well as proteases (7, 26). Microglia are defense cells in the CNS, but over-activated microglia can trigger or exacerbate neuroinflammation and related brain diseases (27). Therefore, attenuating microglial overactivation and its subsequent cellular damage may be valuable therapeutic strategy for neuroinflammation-related brain diseases. Herein, we first reveal that TMP ameliorates neuroinflammation in BV2 cells through activating SIRT1 and inhibiting NF-κB signals.

Neuroinflammation leads significant morphological changes in CNS diseases. The structure of the cortex and hippocampus is pathologically altered, accompanied by inflammatory cell infiltration, altered neuronal morphology and serious neuronal loss in the brain tissue of LPS-induced mice and AD mice (28, 29). In this study, we focused on Sp5 region and found that TMP alleviated neuron loss and morphological changes, including reducing nuclear consolidation, deep staining and eosinophilic cytoplasmic degeneration in the Sp5 region of LPS-induced mice.

Microglia act as the first line of defense to pathogens in CNS, that initiate the immune response and subsequently release a variety of inflammatory mediators (30). Depending on the type of stimulus, microglia polarize into different phenotypes with different functions, mainly including M1 state and M2 state (31). In general, the M1 state can be triggered by LPS and generate a diversity of pro-inflammatory mediators (32). In this study, LPS-induced M1 polarization resulted in a significant rise in TNF-α and IL-6 secretion, and mRNA transcription of COX-2, iNOS, TNF-α and IL-6, while TMP treatment reversed M1 polarization *in vitro*. Moreover, *in vivo*, TMP treatment similarly inhibited M1 microglial polarization, with the decrease of M1 marker, including COX-2, iNOS and CD86, and promoted M2 polarization, with the increase of M2 markers, such as Arg-1 and CD206, and further suppressed pro-inflammatory cytokines and chemokines expressions. Collectively, TMP inhibited LPS-induced M1 polarization, and promoted the polarization of M2 microglia.

NF-κB signaling pathway is an important regulator for various physiological and pathological inflammatory responses (33). Notably, p65 activity is tightly regulated by its inhibitory molecule, IκBα. In the cytoplasm, p65 interacts with the IκB inhibitory family and is activated by scavenging IκB proteins and translocating p65 to the nucleus (34). In the upstream signaling cascade of NF-κB, the IKK complex induces phosphorylation of the IκBα inhibitory protein, leading to its degradation by the proteasome (35). Later, the interaction between IκBα and p65 is disrupted, and then the p65 is transferred from the cytoplasm to the nucleus, which initiates the transcription of pro-inflammatory genes (36). Inhibiting the activation of NF-κB signals in microglia and neurons observably mitigate neuroinflammation in brain disease, manifested by down-regulation of pro-inflammatory mediators, such as IL-6, IL-18, iNOS and COX-2, and the loss of neurons (37–39). In this study, TMP suppressed the phosphorylation of IKKα/β, IκBα and p65 proteins, inhibited the translocation of p65 to nucleus, led a decrease in pro-inflammatory cytokines of LPS-induced neuroinflammation.

SIRT1, a NAD-dependent histone deacetylase, exerts neuroprotective role in Parkinson’s Disease, Huntington’s disease and other brain diseases (40–42), through targeting histones and crucial transcription factors such as NF-κB, thereby inhibiting the transcription and translation of various inflammatory factors (43). Research shows that the expression and activity of SIRT1 are decreased in acute neuroinflammatory response (44). Lack of SIRT1 remarkably activates the NF-κB signals and increases the expressions of pro-inflammatory mediators in LPS-induced microglia (23), and also aggravates neurocognitive impairment and the production of TNF-α and IL-1β in LPS-stimulated brain tissue of mice (44). Consistently, our data showed SIRT1 levels were decreased in BV2 cells and Sp5 region from mice after LPS stimulation, which supports SIRT1 represents an anti-inflammatory target. TMP treatment increased the protein expression of SIRT1 and inhibited the activation of NF-κB pathway, thereby reducing the transcription and translation of downstream pro-inflammatory mediators, which were further confirmed by SIRT1 inhibitor EX527.

## 5 Conclusion

**Figure 8:**
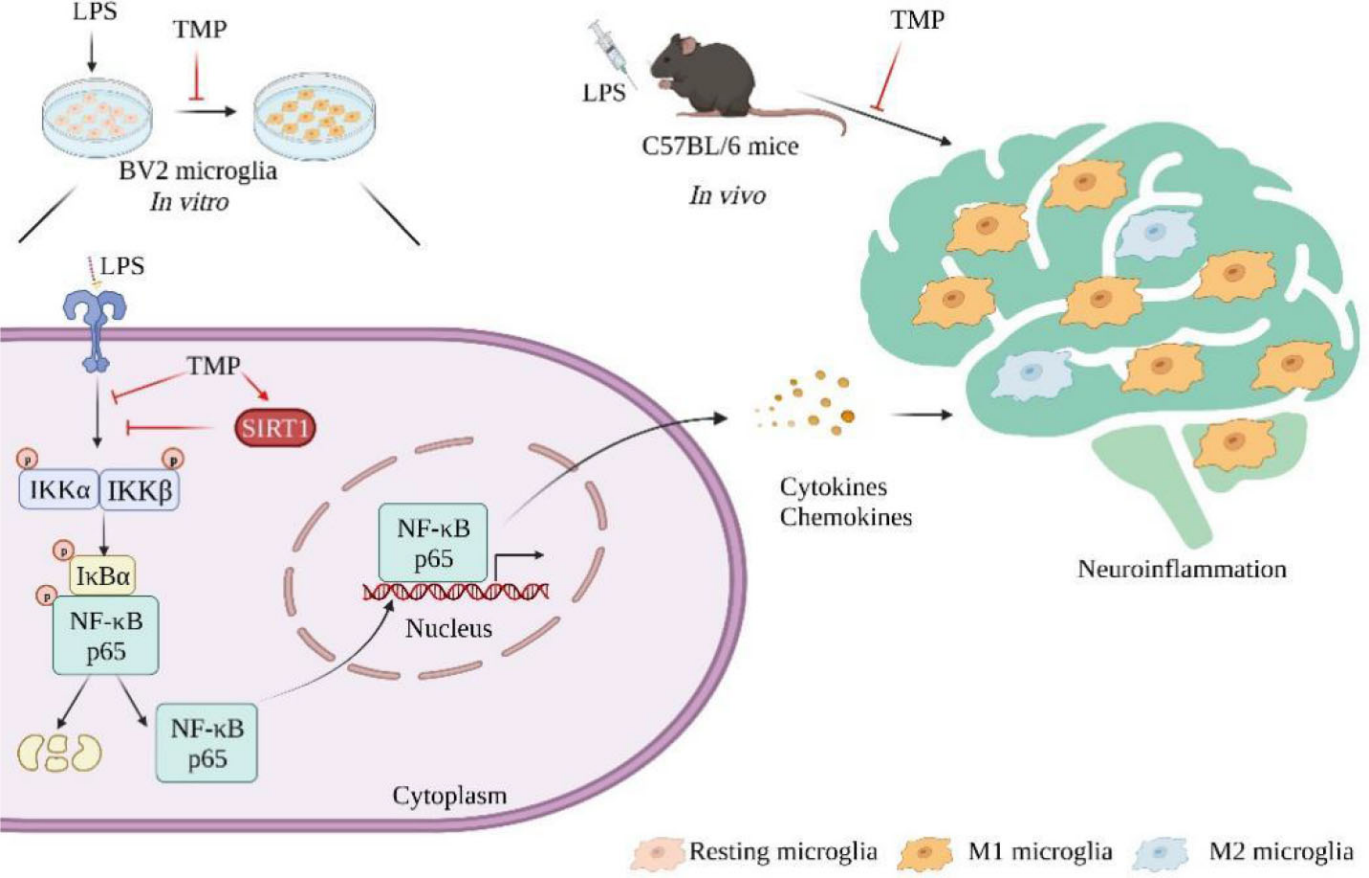
Schematic models of molecular targets of TMP in neuroinflammation. Neuroinflammation is characterized by the secretion of inflammatory mediators and the over-activation of microglia. Therapeutically, TMP exert anti-neuroinflammatory effects through activating SIRT1 and inhibiting NF-κB signaling pathway.

In summary, TMP, an alkaloid derived from *Ligusticum chuanxiong*, attenuates neuroinflammatory responses *in vitro* and *in vivo*, possibly *via* activating SIRT1 and suppressing NF-κB pathway (**Figure 8**), thus indicating TMP might act as therapeutic agent for the treatment of neuroinflammation and associated brain disorders.

## Data availability statement

The original contributions presented in the study are included in the article and further inquiries can be directed to the corresponding author.

## Ethics statement

The animal study was reviewed and approved by the Animal Ethics Committee of Chengdu University of Traditional Chinese Medicine (No. 2021-30). Animal studies were performed in accordance with the Guide for the Care and Use of Laboratory Animals.

## Author contributions

YC, FP and CY performed the experiments, drafted and revise the manuscript. HH and ZX analyzed the results and revise the manuscript. JC and LL analyzed the results. CP and DL conceived of the study, participated in its design and coordination, provided critical feedback and helped shape the manuscript. All authors contributed to the article and approved the submitted version.
